# Method-specific beliefs associated with the choice of future contraception among women in refugee settlements in Uganda

**DOI:** 10.3389/frph.2026.1731971

**Published:** 2026-03-04

**Authors:** George Odwe, Peter Kisaakye, Yohannes Dibaba Wado, Stella Muthuri, Dagim Habteyesus, Gloria Seruwagi, Yadeta Dessie, Bonnie Wandera, Caroline W. Kabiru, Chi-Chi Undie, Francis Obare

**Affiliations:** 1 Population Council, Nairobi, Kenya; 2 African Population and Health Research Center (APHRC), Nairobi, Kenya; 3 Population Council Inc, Nairobi, Kenya

**Keywords:** intentions to use contraception, method-choice, method-specific beliefs, women in refugee setting, Uganda

## Abstract

**Introduction:**

Factors underlying reproductive decisions, including contraceptive method choice, are poorly understood, especially in humanitarian settings where sexual and reproductive health (SRH) needs may be highest due to heightened risk of sexual violence and disruptions of health services. The study examined the association between method-specific beliefs and future method choice among women in refugee settlements in Uganda.

**Methods:**

Data were from a baseline of a one-year prospective study involving a cohort of 2,498 women aged 15–45 years living in Kiryadongo and Kyangwali refugee settlements. Analysis used cross-tabulation with chi-square test and conditional logistic regression analysis to examine associations between method-specific beliefs and intention to use injectables, implants, or pills among contraceptive non-users.

**Result:**

Among contraceptive non-users (*n* = 1,486), 32% intended to use a method within the next 12 months or later. Injectable was the most preferred future method (39%), followed by implants (25%) and pills (17%). Concerns about interference with menstruation, unpleasant side effects, and safety for long-term use were common across all three methods (range 58% – 90%). The likelihood that a woman intended to use injectable, implant, or pill in future was positively associated with perceived ease to access (AOR = 1.95; 95% CI: 1.03–3.66), ease of use (AOR = 4.17; 95% CI: 2.22–7.86), safety for long use (AOR = 4.51; 95% CI: 1.61–12.64), and satisfaction with past use (AOR = 2.87; 95% CI: 1.51–5.46).

**Conclusion:**

Intention to use contraception in future among non-users in refugee settlements is low, coupled with widespread negative beliefs about available methods. There is need to improve counseling to counter negative beliefs and to expand access to a range of modern contraceptive methods.

## 1 Introduction

Globally, an estimated 121 million unintended pregnancies occurred annually between 2015 and 2019, with the highest rates in sub-Saharan Africa (1). The extent and consequences of unintended pregnancy, such as unsafe abortion, are likely to be exacerbated in humanitarian settings due to the heightened risk of sexual violence (2, 3). Preventing unintended pregnancy by ensuring the availability of a range of long-acting reversible and short-acting contraceptive methods, including emergency contraception, is a core element of the Minimum Initial Service Package (MISP) (4). However, access to and coverage for contraception services in humanitarian settings remain highly suboptimal due in part to socio-cultural context, and method-related barriers such as contraceptive beliefs (2, 3).

Method-specific beliefs are key in the decision-making process regarding contraception, including intention-to-use and method-choice (4, 5). Intention-to-use contraception significantly impacts contraceptive outcomes such as uptake, adherence, and continuation (6, 7). Furthermore, in line with psychological and behavioral theories, intention-to-use is considered a proxy measure of future demand and self-identified motivations and preferences, as well as the psychosocial processes that shape behavior (8–10). It directly captures women's stated preferences regarding contraception, their perception of pregnancy risk, and their interest in using contraception in the future; hence, it may be useful for family planning programming (11, 12).

Studies show that beliefs about contraceptive methods, such as, perceived effectiveness, convenience (i.e., ease of use and access), health effects concerns (i.e., interference with menses, side effects, and infertility), and safety for long-term use, are associated with intention to use and choice of a future method (13–16). Studies also indicate that satisfaction with past contraceptive use, positive experiences shared within a woman's social network, and approval from a husband or partner significantly influence contraceptive use and method choice (16–18). Sociodemographic characteristics such as age, fertility preferences, parity, family size, and cultural context have also been shown to be associated with future contraceptive method choice (19–22). Most studies on factors associated with women's preferences for certain contraceptive methods are from development settings. However, little remains known about reproductive decisions, including contraceptive method choice in humanitarian settings where SRH needs may be greatest. Evidence from a descriptive study conducted in refugee settlements in Uganda indicates that women's intention to use contraceptive methods in the future is generally low and largely influenced by perceived health-related concerns (23). However, this study did not account for women's prior contraceptive use, which is a critical determinant of subsequent method choice.

Perceptions and experiences with available contraceptive methods, along with the advice provided by service providers, are crucial to fulfilling reproductive rights (24, 25). However, there is limited understanding of how women's opinions, perceptions, and past experiences with contraceptive methods impact future method choice in humanitarian contexts where the risk of unintended pregnancy is heightened due to high cases of sexual violence and disruptions in access to essential SRHR services (26, 27). This lack of evidence hampers the development of evidence-based interventions and strategies to address low contraceptive use and unintended pregnancies, particularly those that consider the unique circumstances of women and girls in these settings. This study examined the association between method-specific beliefs (perceptions of effectiveness, convenience, health effects, long-term safety), satisfaction with past use, social network experiences, and husband/partner approval) and future intention to use injectables, implants, or pills among women not using any method at the time of the survey (non-users). In addition, the study evaluated the relative importance of these factors in relation to the preferred future method among non-users who are aware of pills, injectables, and implants.

## 2 Data and methods

### 2.1 Context

Uganda is the third-largest refugee-hosting country in the world, with an estimated refugee population of 1.7 million as of May 2024 (28). The majority of refugees in Uganda are from South Sudan (55%) and the Democratic Republic of Congo (31%), with smaller numbers from Somalia, Burundi, Rwanda, and other countries. Furthermore, the majority of this population are women and adolescent girls in need of SRH services. Uganda's progressive legal framework grants refugees' access to social services, including health and education (29). Health services, including SRH, are delivered through public and private facilities, supported by the United Nations High Commissioner for Refugees (UNHCR) and its implementing partners.

The study was conducted in two refugee settlements in Uganda: Kiryandongo and Kyangwali. As of July 2024, Kiryandongo hosted approximately 119,129 refugees, mostly from South Sudan (98%), while Kyangwali had about 139,935 refugees, primarily from the DRC (96%) (28). These settlements were selected in consultation with UNHCR-Uganda and the Department of Refugees in the Office of the Prime Minister (OPM), which represent major refugee groups in Uganda.

### 2.2 Study design

The analysis draws on baseline data from a one-year prospective study involving a cohort of randomly selected women and girls aged 15–45 years living in Kiryadongo and Kyangwali refugee settlements. The overall study aims to generate evidence on innovative solutions for addressing unintended pregnancy in refugee settings. The upper age limit of 45 years was chosen to ensure follow-up interviews with the women while within reproductive age.

### 2.3 Sampling

Sampling was in two stages. First, two zones were purposively selected in each of the two settlements based on predefined criteria relevant to the study objectives—specifically, their differing program implementation contexts, population size (about 3,000 to 5,000 households) and accessibility. Household listing was then conducted in the selected zones to generate the sampling frame from which eligible women and girls were randomly selected. A sample size of 3,019 women aged 15–45 was targeted to detect differences of 20%–50% in two proportions at a 95% confidence level and 80% power, based on a formula developed by Fleiss and colleagues (30). Sample size calculations assumed that both exposure/predictor and outcome variables are dichotomous, for example, assuming 20% in the unexposed group and 40% in the exposed group having a positive outcome, such as the current use of pills or injectables to prevent unintended pregnancy. Interviews were completed with 2,498 women (78% of those sampled).

### 2.4 Data collection

Data collection was conducted from March to May 2024. Face-to-face computer-assisted personal interviews (CAPI) were conducted by trained female interviewers with sampled eligible participants using a structured questionnaire. Pre-testing of the tool was done to identify and address any issues prior to the actual data collection. Interviews were conducted in English or five other local languages (Acholi, Juba Arabic, Kigegere, Kinyabwisha and Kiswahili) depending on a participant's preference. All data was electronically recorded on the ODK platform.

### 2.5 Measures

The questionnaire was adopted from a previous study that examined reasons for unmet need for family planning, with attention to the measurement of fertility preferences in low and middle income countries (31). Information captured includes women's sociodemographic characteristics, reproductive history, contraceptive use, and fertility intentions. Participants were asked if they planned to use contraception within the next 12 months or at any time in the future. Women who responded positively were then asked which method they intended to use. The outcome variable for this study was whether women who were not using a method at the time of the interview intended to use a contraceptive method (implants, injectables or pills) in the future.

All women who had heard of a specific method (i.e., implants, injectables, pills, emergency pills and male condoms) were asked about their beliefs about that method with respect to convenience, effectiveness, and method-related health effects, regardless of whether they were using it or not. In terms of convenience, women were asked to state their opinions on whether the method was easy to obtain and easy to use. Perceived effectiveness was determined by asking whether they considered the method “very effective at preventing pregnancy”. Five items regarding method-related health concerns and safety were assessed: women were asked whether they believed the method was likely to cause (a) serious health problems, (b) unpleasant side effects, (c) disruption to regular menses, (d) long-term infertility, or (e) dangers from long-term continuous use. It is worth noting that beliefs (b) and (c) may be experiential when one is using contraception, while beliefs (a), (d), and (e) are perceptions driven by an expectation of a bodily change which is unsupported by current clinical evidence (32). In addition, the questionnaire asked women opinions about their partner's approval of the method while those who reported past used were asked about their level of satisfaction with the method, which was categorized into: past use and satisfied, or otherwise. Additionally, women were asked if their social network (including friends, relatives, and neighbours) had used the three methods and whether their experience had been satisfactory.

### 2.6 Analysis

Descriptive statistics was used to examine the characteristics of all interviewed women and crosstabulation with a Chi-square test to assess method-specific beliefs about three popular methods (pills, injectables, and implants) among non-users aware of these three methods. The analysis also assessed which perceived method-specific attributes predict future intentions to use either injectables, implants, or pills among women who were not currently using a method but intended to do so in the next 12 months or at any time in the future and were aware of all three methods.

Alternative specific conditional logit (McFadden's choice model) was used to assess the association between method-specific beliefs and intention to use either implants, injectables, or pills in the future. This model, commonly used in econometric analysis of choice (33), allows the inclusion of two types of independent variables in one regression equation: alternative-specific and case-specific (34). The alternative-specific independent variables (method-specific beliefs) vary among alternatives (methods) as well as cases (respondents). On the other hand, the case-specific independent variables (respondents background characteristics) vary only among cases (respondents). The model relies on the key assumption of Independence of Irrelevant Alternatives (IIA), that is, the relative probability of choosing one alternative over another remains constant in the presence or absence of other alternatives (35, 36). However, this assumption may not hold in some contexts, leading to biased estimates (37). In this paper, each method attribute or belief is represented by a single coefficient indicating its association with future method choice. For example, one might consider whether, after accounting for other factors, women are less likely to choose a method perceived to have specific health effects, such as interfering with menstruation. For respondents' characteristics, arrays of coefficients are provided for effects on two pairwise method choices: implant vs. injectable and pill vs. injectable. A similar approach has been used in published studies (13, 14). A *p*-value < .05was used to indicate the statistical significance with 95% confidence intervals. Estimation was via maximum likelihood using the *asclogit* command in Stata version 16.

### 2.7 Ethical considerations

The Population Council Institutional Review Board (Protocol 1027) and the Mildmay Uganda Research Ethics Committee (MUREC) (REF 0109–2023) granted ethical approval for the study. The research was also granted regulatory approval by the Uganda National Council for Science and Technology (REF SS2085ES). All participants provided consent, electronically recorded on the ODK platform.

## 3 Results

### 3.1 Background characteristics

Out of 2,264 women who completed the interview, 45% and 55% were from Kiryandongo and Kyangwali refugee settlements, respectively (Table 1). Nearly half of the women were aged 25–34 years (44%) and had no formal education (46%). About 60% were married or living with a partner, 28% did not want to have (another) child and 11% were pregnant at the time of the interview. Only 21% were currently using any method. More than half (55%) of the respondents were originally from the Democratic Republic of Congo (DRC), while 44% were from South Sudan, and the rest were from other countries. A significantly higher proportion of current users than non-users were aged 25–34 years, had primary or higher level of education, and wanted a child but preferred to wait 2–5 years.

**Table 1:** Percentage distribution of respondents by background characteristics and constraceptive use status.

Background characteristics	Using (*N* = 469)	Not using (*N* = 1,795)	All (*N* = 2,264)	*p*-value
Age				
15–24	27.3	25.5	25.9	
25–34	48.8	42.5	43.8	0.002
34–45	23.9	32.0	30.3	
Educational attainment				
No education	37.1	48.3	46.0	
Primary incomplete	32.6	33.3	33.2	<0.001
Primary complete/some	22.2	15.8	17.1	
Secondary complete +	8.1	2.6	3.8	
Marital status				
Not married/living with a man	30.9	43.3	40.8	<0.001
Married/living with a man	69.1	56.7	59.2	
Fertility preference				
Want a child: soon/within 2 years/undecided	11.5	14.4	13.8	
Want a child: wait 2–5 years	50.3	30.2	34.4	
Want a child: wait 5+ years	11.9	23.1	20.8	<0.001
Does not want a/another child/sterilized	26.2	32.3	31.0	
Country of origin				
South Sudan	46.7	42.9	43.7	
DRC	51.2	56.0	55.0	0.058
Others	2.1	1.1	1.3	
Settlement				
Kiryandogo	49.3	44.1	45.2	
Kyangwali	50.8	55.9	54.8	0.047
Total	20.7	79.3	100.0	

Among contraceptive nonusers, 96% were neither infertile nor postmenopausal (Table 2). Of these, 32% intended to use a contraceptive method within the next 12 months or later. Among those intending to use contraception, 39% preferred injectables, 25% implants, 17% pills, 9% other modern methods, 6.6% traditional methods, and 4% were undecided.

**Table 2:** Percentage distribution of contraceptive nonusers by selected characteristics.

Characteristic	*N*	%
Currently pregnant	(*N* = 1,795)	
Yes	247	13.8
No	1,548	86.2
Unable to become pregnant/	(*N* = 1,548)	
Yes	62	4.0
No	1,486	96.0
Intends to use a method of contraception	(*N* = 1,486)	
Yes, in the next 12 month	304	20.5
Yes, later on	170	11.4
No	926	62.3
Do not know/unsure	86	5.8
Preferred method of contraception	(*N* = 474)	
Implant	120	25.3
Injectable	185	39.0
Pill	79	16.7
Other modern methods	41	8.7
Traditional methods	30	6.3
Do not know/unsure	19	4.0
Intends to use injectables, implants, or pills and aware of all three methods^a^	263	55.5

a -Of the 474 women who were not currently using a method and intended to use any method in the future, 263 women intended to use either injectables, implants, or pills and knew all three methods.

### 3.2 Perceived contraceptive attributes

Table 3 summarizes the beliefs about pills, injectables, and implants among non-users who are fecund, have heard of all three methods, and intend to use one of these methods in the next 12 months or later (*n* = 263). The proportion of women who believed that injectables are easy to obtain, effective, and easy to use was significantly higher than for implants and pills (range 76%–89%).

**Table 3:** Method-specific beliefs among nonusers who reported knowing about pills, injectables and implants.

Attributes	Implant		Injectable		Pills		*p*-value
	n	%	n	%	n	%	
Perceived convenience and effectiveness							
Easy to obtain	180	68.4	231	87.8	204	77.6	<0.001
Effective at preventing pregnancy	219	83.3	230	87.5	194	73.8	<0.001
Easy to use	154	58.6	201	76.4	149	56.7	<0.001
Health effects beliefs							
Causes serious health problems	77	29.3	46	17.5	25	9.5	<0.001
Interfere with menstruation	153	58.2	152	57.8	101	38.4	<0.001
Causes unpleasant side effects	200	76.1	164	62.4	162	61.6	<0.001
Unsafe for long-term use (without a break)	241	91.6	251	95.4	249	94.7	0.115
Cause infertility	69	26.2	45	17.1	59	22.4	0.040
Husband approval and social network experiences							
Husband approves of method	66	25.1	71	27.0	66	25.1	0.868
Have a friend/relative/neighbor who has used the method	180	68.4	189	71.9	143	54.4	0.005
Friends/relatives/neighbors are satisfied with the method^b^	153	85.0	175	92.6	129	90.2	0.057
Past use and satisfaction	18	6.8	30	16.7	17	14.5	<0.001
Total women (N)^a^	263	100.0	263	100.0	263	100.0	

a N includes women aware women who intended to use either injectables, implants, or pills, and who knew all three methods.

b This panel only includes the women who had a social network who had used the method.

There were significant differences in perceived health-related concerns by type of method. Concerns about unspecified serious health problems were highest for implants (29%) and injectables (18%), compared to pills (10%). Similarly, concerns about interference with menstruation were highest for implants and injectables (both at 58%) compared to pills (38%). Concerns about unpleasant side effects were common across all three methods (range 62%–76%). The majority of women (over 90%) believed that it was unsafe to use implants, injectables, or pills for long-term without taking a break. More than a quarter of the women (26%) believed that implants cause infertility, compared to 17% for injectables and 22% for pills.

The proportion of women reporting knowing someone in their social network who had used the method was significantly higher for injectables (72%) than implants (68%) or pills (54%). Perceived satisfaction among social networks was high across the three methods (range 85%–92%). About 15%–17% of past users of pills and injectables, compared to only 7% of implants, reported satisfaction with the method.

### 3.3 Intention to use injectable, implants, or pills

Table 4 presents the results from the conditional logistic regression analysis of non-users who expressed an intention to use either injectables, implants, or pills in the next twelve months or later. In Model I (unadjusted), all attributes, except for non-interference with menstruation, were associated with future intention to use either injectables, implants, or pills. The belief that a method was easy to use or safe for long-term use had the strongest association with the intention to use either injectables, or implants, or pills. This was followed by beliefs such as the method was easy to obtain, use satisfaction within the social network, absence of serious health problems, and effectiveness in preventing pregnancy (OR range 2.90–3.90), as well as absence of long-term fertility impairment and unpleasant side effects (OR range 1.90–2.30). Satisfied past use was associated with increased odds (OR = 3.88; 95% CI = 2.24–6.72) of intending to use injectables, implants, and pills compared to women who had never used any of these methods. If a woman's husband/partner approved the method, she was four times (OR = 4.17; 95% CI = 1.68–10.35) more likely to indicate an intention to use the method compared to her counterparts whose husbands/partners disapproved the method.

**Table 4:** Results from the conditional logistic regression model showing the odds of future intention to use injectables, implants among non-users and pregnant women who know all three methods.

Method Choice: Injectable, Implant, or Pill	Model I		Model II	
	Crude OR [95% CI]	*P*>|z|	AOR [95% CI]	*P*>|z|
Method beliefs				
Easy to obtain	3.89 [2.23,6.76]	0.000	1.95 [1.03,3.66]	0.039
Effectively prevents pregnancy	2.94 [1.68,5.13]	0.000	1.53 [0.74,3.16]	0.247
Easy to use	6.57 [3.96,10.90]	0.000	4.17 [2.22,7.86]	0.000
Absence of serious health problems	3.00 [1.81,4.98]	0.000	1.49 [0.78,2.86]	0.227
No interference with menstruation	1.42 [0.96,2.10]	0.082	1.37 [0.82,2.29]	0.223
Absence of unpleasant side effects	1.93 [1.28,2.93]	0.002	0.95 [0.60,1.52]	0.846
Safe for long-time use (without a break)	6.11 [2.77,13.46]	0.000	4.51 [1.61,12.64]	0.004
No long-term fertility impairment	2.27 [1.25,4.12]	0.007	0.86 [0.41,1.81]	0.690
Social network tried and satisfied	3.03 [2.06,4.45]	0.000	1.59 [0.88,2.87]	0.121
Partner's approval				
Approves	4.17 [1.68,10.35]	0.002	2.18 [0.77,6.16]	0.140
No husband	0.61 [0.19,1.89]	0.388	0.56 [0.12,2.48]	0.442
Past use and satisfaction (Ref: Never used)				
Past user and satisfied	3.88 [2.24,6.72]	0.000	2.87 [1.51,5.46]	0.001
Past user and dissatisfied/mix/neither	0.80 [0.41,1.56]	0.511	0.98 [0.40,2.38]	0.962
Injectable (Reference group)				
Effects on Choice of Implant (vs. Injectable)				
Age group (Ref: 15–24 years)				
25–34 years	1.22 [0.64,2.36]	0.544	0.78 [0.34,1.79]	0.552
35–45 years	1.64 [0.73,3.67]	0.230	1.72 [0.57,5.16]	0.334
Educational attainment (Ref: no education)				
Primary incomplete	0.57 [0.29,1.10]	0.093	0.43 [0.19,1.02]	0.056
Primary complete/some secondary	1.38 [0.63,3.00]	0.417	1.63 [0.56,4.75]	0.375
Secondary complete +	0.73 [0.20,2.62]	0.625	0.27 [0.04,1.65]	0.155
Fertility Preference (Ref: Want to soon/want within 2 years/undecided)				
Want to wait 2–5years	1.39 [0.53,3.69]	0.505	1.19 [0.35,4.04	0.784
Want to wait 5+ years	0.83 [0.25,2.76]	0.763	0.49 [0.11,2.20]	0.349
Want no more	1.75 [0.62,4.96]	0.291	1.31 [0.344.98]	0.692
Settlement				
Kiryandogo				
Kyangwali	0.71 [0.40,1.27]	0.250	0.58 [0.25,1.32]	0.194
Effects on Choice of Pill (vs. Injectable)				
Age group (Ref: 15–24 years)				
25–34 years	1.13 [0.56,2.27]	0.735	1.21 [0.50,2.94]	0.679
35–45 years	0.64 [0.22,1.83]	0.402	0.93 [0.24,3.65]	0.923
Educational attainment (Ref: no education)				
Primary incomplete	0.42 [0.20,0.89]	0.023	0.33 [0.12,0.86]	0.023
Primary complete/some secondary	0.83 [0.34,2.04]	0.690	0.77 [0.23,2.58]	0.674
Secondary complete +	0.42 [0.08,2.11]	0.290	0.20 [0.02,1.66]	0.135
Fertility Preference (Ref: Want to soon/want within 2 years/undecided)				
Want to wait 2–5years	1.42 [0.51,3.95]	0.506	1.15 [0.35,3.78]	0.819
Want to wait 5+ years	0.97 [0.28,3.35]	0.961	0.48 [0.11,2.05]	0.319
Want no more	0.53 [0.15,1.93]	0.337	0.39 [0.08,1.82]	0.230
Settlement				
Kiryandogo				
Kyangwali	1.27 [0.64,2.52]	0.494	0.69 [0.27,1.78]	0.444

OR, odds ratios; AOR, adjusted odds ratios.

In Model II (adjusted), only four attributes remained significantly associated with the intention to use injectables, implants, or pills in the next twelve months or later. Women who perceived the method to be easy to obtain or easy to use had 1.95 (95% CI = 1.03–3.66) times and 4.17 (95% CI = 2.22–7.86) times higher odds, respectively, of indicating an intention to use a method. Similarly, women who perceived the method as safe for long-term use (without a break) had higher odds (OR = 4.51; 95% CI = 1.61–12.64) of indicating an intention to use a method compared to those who perceived otherwise. The odds of choosing that method in future increased if members of the woman's social network were satisfied users of any of the methods (OR = 1.94; 95% CI = 1.18–3.19) compared with women with no or whose social network tried but had an unsatisfactory experience with the method. Additionally, women who had ever used a method and were satisfied had increased odds (OR = 2.87; 95% CI = 1.51–5.46) of choosing the method in future than women who had never used the method.

The lower panels of Table 4 display the effects of respondent characteristics on method choice, first comparing the choice of implant vs. injectable and then the choice of pill vs. injectable. Notably, there were no statistically significant differences in the choice of implants or pills over injectables based on age, fertility preference, and settlement. However, the odds of choosing pills over injectables were reduced for women with incomplete primary education compared to those with no education.

## 4 Discussion

Factors underlying reproductive decisions, including contraceptive method choice, are poorly understood, especially in humanitarian settings where SRH needs are heightened due to increased risks of sexual violence and disruptions to access to essential health services (38). This study examined method beliefs associated with future choice for implants, injectables, or pills among women aged 15–45 years in refugee settlements in Uganda who were non-users of these methods. The study contributes to the literature on method-specific beliefs associated with future contraceptive choices and could inform the design of effective contraceptive counselling interventions in humanitarian settings.

Result shows a high contraceptive nonuse among women aged 15–45 years in two refugee settlements (80%). Despite half of the women interviewed preferring to delay having a child for at least 5 years or stop childbearing altogether, only a third of nonusers (32%) expressed intention to use a method in the future. Intention to use contraceptives is substantially lower among women of reproductive age in refugee settings than in development settings. For example, analysis of the 2016 Uganda Demographic and Health Survey found that 60.2% of non-users in development settings intended to use contraceptives in the future (12). Additionally, 81% of current non-users who intend to use contraception in the future specified injectables, implants, or pills as their preferred methods. Notably, the rank order of preference among these three methods mirrors their relative popularity in Uganda (39).

Low intention to use contraceptives was coupled with unfavorable beliefs. Most women (92%–95%) believed that prolonged use of injectable, implant, or pills without a break was unsafe, and many reported that using these three methods would cause unpleasant side effects. Additionally, more than half of the women believed that using implants or injectables would interfere with menstruation, and a significant proportion (between 17%–26%) believed that using any of the three methods could cause infertility. Thus, many women in refugee settings are concerned about the health effects of contraception, which may affect current and future use. The findings are consistent with results from studies based in urban informal settlements and rural Kenya, highlighting the anxiety women have about the impact of hormonal contraceptives on their health and the anticipation of side effects (14, 15). Similarly, a study in rural Matlab revealed that a woman's intention to use a method was positively associated with her perception that it is easy to use, does not cause serious health problems, and does not affect long-term fertility (13).

Perceived convenience (ease of obtaining or using the methods) was significantly associated with intention to use injectable, implant, or pills in the future. This finding highlights the need to improve contraceptive availability and accessibility in these settings. More efforts should focus on increasing the number of service delivery points offering family planning methods, expanding the number of trained providers, improving the range of available methods, and addressing contraceptive security (40). A potential strategy to improve access to family planning services is to integrate contraceptive provision with other community-based initiatives such as food distribution and refugee verification platforms. Leveraging existing community platforms creates a strategic entry point for delivering FP information and services (41, 42).

Perceived safety for longer-term use was positively associated with future intention to use either injectables, implants, or pills, even after controlling for other attributes. While there is no clear explanation for this association, notably that over 90% of women in the study considered the use of injectables, pills, and implants unsafe for extended periods without taking breaks, reflecting their anxiety about potential health effects (43).

Social networks and past experiences with these methods also played a significant role in shaping future intention to use injectables, implants, or pills. Women who had used these methods in the past or whose social networks had tried these methods and were satisfied were more likely to express an intention to use these methods in the future. A woman's social network influences her decision to choose and adopt a particular method (44, 45). The findings align with evidence from other settings. For instance, Mumah et al. found that perceived method-specific attributes, including women's own past experiences with contraceptive methods and those of their friends, predicted preferred future contraceptive choices among 317 women living in Nairobi informal settlements who were not currently using any method but intended to start using injectables, implants, or pills within the next 12 months (14). Another study by Tolla and colleagues among adolescent girls and young women (AGYW) living in resource-constrained settings in Cape Town, South Africa, found that interpersonal networks play a critical role in shaping AGYW's attitudes toward contraception, as well as in motivating or discouraging their use of and access to contraceptive services (16).

Satisfied past users of a method were more likely than never-users to report an intention to use injectables, pills, or implants. The findings corroborate the results from a study among women in urban informal settlements that showed that satisfaction with past use was associated with the choice of a future method (14). Personal experience, particularly satisfaction among past users, has a positive influence on future intentions to use the same method (46, 47), suggesting that positive firsthand experience builds confidence, reduces uncertainty, and reinforces trust in the method's effectiveness and suitability.

### 4.1 Limitations

The study has some limitations. The data analyzed is based on women's self-reports, which may introduce reporting biases, such as cognitive and social desirability. The sensitive nature of questions about contraceptive use may lead to underreporting or overreporting of beliefs. Although it would have been ideal to examine the entire spectrum of contraceptive methods, the analysis was limited to three methods (pills, injectables, and implants). The study did not analyse other methods due to insufficient number of cases for meaningful statistical analysis. Despite these limitations, the study is innovative in its detailed measurement of method-specific perceptions that may influence future decisions to adopt or continue using specific methods among women in humanitarian settings. Additionally, there are plans to investigate further how women's opinions or stated intentions about contraceptive choice affect actual method use based on contraceptive calendars to be captured during follow-up interviews.

### 4.2 Conclusion

Intention of contraception among non-users in refugee settlements is low, with widespread negative perceptions of available methods. Future method choice is shaped by perceived convenience, long-term safety of the method, social network satisfaction, and past experiences. These findings highlight the need to improve counseling to counter unfounded negative beliefs and to expand access to a range of contraceptive methods.

## Data Availability

The original contributions presented in the study are included in the article/Supplementary Material, further inquiries can be directed to the corresponding author.
